# High Resolution Magnetic Resonance Imaging for Characterization of the Neuroligin-3 Knock-in Mouse Model Associated with Autism Spectrum Disorder

**DOI:** 10.1371/journal.pone.0109872

**Published:** 2014-10-09

**Authors:** Manoj Kumar, Jeffery T. Duda, Wei-Ting Hwang, Charles Kenworthy, Ranjit Ittyerah, Stephen Pickup, Edward S. Brodkin, James C. Gee, Ted Abel, Harish Poptani

**Affiliations:** 1 Department of Radiology, Perelman School of Medicine at the University of Pennsylvania, Philadelphia, Pennsylvania, United States of America; 2 Department of Biostatistics and Epidemiology, Center for Clinical Epidemiology and Biostatistics, Perelman School of Medicine at the University of Pennsylvania, Philadelphia, Pennsylvania, United States of America; 3 Department of Biology, University of Pennsylvania, Philadelphia, Pennsylvania, United States of America; 4 Center for Neurobiology and Behavior, Department of Psychiatry, Perelman School of Medicine at the University of Pennsylvania, Philadelphia, Pennsylvania, United States of America; The George Washington University, United States of America

## Abstract

Autism spectrum disorders (ASD) comprise an etiologically heterogeneous set of neurodevelopmental disorders. Neuroligin-3 (NL-3) is a cell adhesion protein that mediates synapse development and has been implicated in ASD. We performed ex-vivo high resolution magnetic resonance imaging (MRI), including diffusion tensor imaging (DTI) and behavioral (social approach and zero maze) tests at 3 different time points (30, 50 and 70 days-of-age) on NL-3 and wild-type littermates to assess developmental brain abnormalities in NL-3 mice. MRI data were segmented in 39 different gray and white matter regions. Volumetric measurements, along with DTI indices from these segmented regions were also performed. After controlling for age and gender, the NL-3 knock-in animals demonstrated significantly reduced sociability and lower anxiety-related behavior in comparison to their wild type littermates. Significantly reduced volume of several white and gray matter regions in the NL-3 knock-in mice were also observed after considering age, gender and time point as covariates. These findings suggest that structural changes in the brain of NL-3 mice are induced by the mutation in the NL-3 gene. No significant differences in DTI indices were observed, which suggests that the NL-3 mutation may not have a profound effect on water diffusion as detected by DTI. The volumetric and DTI studies aid in understanding the biology of disrupting function on an ASD risk model and may assist in the development of imaging biomarkers for ASD.

## Introduction

Autism spectrum disorder (ASD) comprises a complex and etiologically heterogeneous group of neurodevelopmental disorders with an unknown unifying pathogenesis. While ASD is defined by the presence of deficits in social interaction as well as restricted and repetitive patterns of behavior, ASD is nevertheless highly heterogeneous in terms of behavior and genetic abnormalities [1]–[5]. The etiology of ASD is unknown in most cases, but monogenic heritable forms exist that have provided insights into ASD pathogenesis and have led to the notion of autism as a ‘synapse disorder’ [4]. Neuroligins are a family of postsynaptic cell-adhesion molecules that are ligands for neurexins, a class of synaptic cell-adhesion molecules [6], and Jamain et al. [7] reported an inherited mutation in the Neuroligin-3 (NL-3) gene in a family with two brothers having ASD. The point mutation at the amino acid position 451 (R451C) caused a decrease in the amount of NL-3 in patients with ASD. Later, the same point mutation was introduced in a mouse to generate the NL-3 R451C knock-in mouse model of ASD [6]. The NL-3 knock-in mice have been reported to exhibit behavioral symptoms similar to those observed in human ASD [6]. However, a subsequent study did not observe behavioral phenotypes relevant to ASD in this model, and therefore raised concerns about the relevance of the model to ASD [8].

Magnetic Resonance Imaging (MRI) has been employed extensively to examine morphological changes in human ASD [9]–[11] as well as in mouse models relevant to ASD [2], [3], [12]–[14]. More recently, diffusion tensor imaging (DTI) has also been used to characterize ASD in humans [15]–[17] and animal models [2], [3], [12], [14]. High resolution microscopic MRI studies have been used to characterize inbred (BALB/cJ, and BTBR) [12], [14] as well as genetic models of ASD (NL-3 knock-in, 16p11.2) [3], [13], [14], [18]. These studies have been primarily performed on isolated fixed brain specimens and have reported volumetric differences in several gray and white matter regions of the ASD brain relative to normal controls [3]. Recently a DTI study also reported a correlation between social abnormalities and fractional anisotropy (FA) in the pre-pubescent BALB/cJ mouse model [12].

High resolution MRI studies of the adult NL-3 knock-in mouse brain (108 day-old) indicated reduced volume in several regions of the brain, compared to wild-type (WT) littermates. Despite, volumetric differences, no differences were noted in any of the DTI parameters in this study [3]. Since reduced interest in social interaction (low sociability) is especially prominent in childhood ASD and the BALB/cJ mice exhibit low sociability and DTI abnormalities at a pre-pubescent age [2], we hypothesized that the lack of differences in DTI in the previous study of NL-3 mice may have been due to the adult age of the mice, as well as the smaller sample size used [3]. Thus in the current study behavioral assays (social approach and elevated zero maze tests) and high resolution MRI studies were performed on 28–30 (juvenile), 48–50 (peri-pubescent) and 68–70 (early adult) days old NL-3 knock-in mice in order to characterize the developmental brain changes in this model and to also look for correlations between behavior and MRI findings.

## Methods

### Animal housing and breeding

A breeding pair of NL-3 knock-in mice (JAX strain number 008475) was procured from The Jackson Laboratory (Bar Harbor, ME), bred in-house at the University of Pennsylvania, and offsprings were genotyped using protocols from Jackson Labs. All animal experiments were approved by the Institutional Animal Care and Use Committee (IACUC). At 2–4 days postnatally, litters were culled to two males and two females to ensure adequate nutrition for all pups and a more uniform social environment during development and to also ensure that litters were balanced in the numbers of males and females. Mice were group housed in a light and temperature controlled animal facility that is accredited by the Association for Assessment and Accreditation of Laboratory Animal Care. Males were removed from breeding cages prior to the birth of pups. All efforts were made to minimize animal pain and discomfort. Water and standard rodent chow were available *ad lib*. With the exception of a weekly cage change, mice were not handled until after the completion of all behavioral tests. To avoid any confounding effects, similar conditions for housing, feeding and handling were used throughout the study. This study was started during the spring of 2011 (4/2011) and completed by in the fall of 2011 (10/2011). A few animals were randomly selected after each time point (30, 50 and 70 day-of-age) for imaging. Details of the animal distributions including age, gender and strains for behavioral (social approach and elevated zero maze test) and imaging studies are summarized in Table S1. Due to the longitudinal nature of the study, some of the animals underwent behavioral study more than once but since the behavioral studies were separated by 3-weeks, any potential effects of repeated behavioral testing were minimized. Seasonal effects and cohort differences were further minimized by selecting the wild type and mutant animals from the same litter to randomize these effects between the groups. To reduce the effect of handling, only one person (R.I.) performed all behavioral assays throughout the study.

### Social approach and anxiety tests

Social Approach Test (also known as the Social Choice Test) was performed on NL-3 knock-in mice and WT mice at 28 (juvenile), 48 (peri-pubescent) and 68 (early adult) days-of-age using a 3-chambered Plexiglas apparatus under dim lighting (<2 lux) during testing in order to minimize the general stress level of the mice, as described previously [2], [12], [19], [20]. The social approach test was performed in the morning between 9:00 AM to 12:00 noon and all procedures were videotaped by using a Sony digital video camera with Night Shot (infrared) feature for recording in low light. The amount of time spent sniffing the stimulus mouse was used as the outcome variable for statistical analysis, with longer sniffing time indicating higher sociability.

Anxiety-related behavior was also assessed in these animals using the elevated zero maze test, which was performed a day after the social approach test (at 29, 49 and 69 days-of-age) at approximately the same time of the day. A circular variant of the elevated plus maze, the zero maze apparatus consists of a raised circular track divided into two open and two closed quadrants. The track had an internal diameter of 40.5 cm and a width of 5.1 cm and was elevated off the floor at a height of 40 cm. The closed quadrants had walls that were 11 cm high. The animal was placed into the center of a closed quadrant and observed for 5 min. Digitized video of each 5 min trial was scored manually to determine the total time spent in open and closed quadrants and the number of transitions between each open and closed quadrant [21], [22]. Longer time spent in the open quadrants indicates lower anxiety-related behavior.

After completion of the zero maze tests, a subset of NL-3 knock-in mice [n = 25 (10 at 29 day-old, 10 at 49 day-old and 5 at 69 day-old)] and WT littermates [n = 24 (7 at 29 day-old, 9 at 49 day-old and 8 at 69 day-old)] were sacrificed at each time point to perform high resolution MRI on extracted brain samples. Because we randomly selected a subset of animals after the zero maze test for imaging, not all animals had behavioral data at all three time points. The distribution of age, gender and strain of all animals used in this study is summarized in Table S1. The animals were anesthetized with an intraperitoneal injection of ketamine. Following lack of deep tendon responses, the thoracic cavity was opened under aseptic conditions and the animals were perfused through the left ventricle with phosphate saline buffer (PBS), followed by 4% paraformaldehyde (PFA) solution. After perfusion, the brain was removed from the skull. The extracted perfused brains were stored at 4°C in 4% PFA solution for 2 weeks to fix the brain tissue prior to ex vivo high resolution DTI studies.

### Sample preparation for *ex vivo* high resolution DTI

Prior to imaging, brain samples were removed from the fixative and switched back to PBS solution for 48 hours at 4°C to rehydrate the tissue. After 48 hours, the sample was removed and rinsed twice with fresh PBS solution and blotted using a soft tissue paper to remove any remaining PBS. The brain was then placed in a plastic tube filled with proton-free susceptibility-matching fluid (Fomblin, Ausimount, Thorofare; USA).

### *Ex vivo* high resolution DTI

High resolution DTI was performed on a 9.4T, 8.9 cm vertical bore magnet equipped with a 55 mm inner-diameter 100 gauss/cm gradient tube and interfaced to a Direct Drive console (Agilent, Palo Alto, CA, USA) running the vnmrj 2.3.C software version. All MRI studies employed a horizontally mounted 2 turn, 11×20 mm (ID x length) solenoid RF coil for transmit and receive. The plastic tube containing the brain sample was placed inside the coil and the probe was mounted inside the magnet. Scout images were acquired in 3 orthogonal planes to localize the position and orientation of the brain sample. DTI data was then acquired using a 3D multi-echo pulsed-gradient spin echo sequence [23] with oval sampling in the two phase encoding directions. Acquisition parameters were: TR  = 800 ms; TE  = 29.50 ms; number of echoes  = 6, number of averages  = 1, b-value  = 902 mm^2^/s, FOV (field of view)  = 17 mm×8.5 mm×10 mm; acquisition matrix size = 136×68×80, resulting in a 125 µm isotropic resolution. The diffusion-weighted images were acquired with diffusion weighting in 6 non-collinear directions with both positive and negative diffusion gradient amplitudes in addition to 2 reference scans with minimal diffusion weighting for a total of 14 data sets resulting in a total acquisition time of 13 hours and 19 minutes per sample.

### DTI data processing and quantification

#### Image processing

Image reconstruction was performed offline using in-house custom software developed in the IDL programming environment (ITT Visual Information Solutions, Boulder, CO, USA). Following application of a Gaussian filter and 3D Fourier transformation, the magnitude images from each echo train were summed in order to improve SNR. Data sets acquired with opposite polarity diffusion weighting gradients were then combined in order to minimize contributions of background gradients to the diffusion data [24]. The resulting group of images was saved in DTI studio [25] format for further processing.

**Figure 1:**
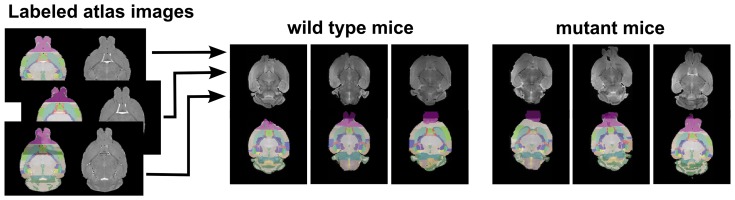
Demonstrating co-registration and segmentation approaches for the analysis of MRI images from the mouse brain. Each of the manually labeled brain was co-registered to each sample's B0 image. The corresponding labels (illustrated as a semi-transparent color overlay) were warped into the sample space and merged via STAPLE. Resulting anatomical labels are illustrated for single wild type and mutant type samples. The olfactory bulb was not included in this analysis due to the variability in extracting the brain tissue leading to incomplete extraction of the olfactory bulbs in some cases. While the resulting labels were probabilistic, the hard segmentations seen here were used for visualization and quality control, and were created by assigning the label of highest probability at each voxel. The color indicates the different regions of the brain.

The b = 0 images for each mouse were registered to a general brain template in preparation for automated region based segmentation such that the same ROI was assessed from each specimen. The alignment and segmentation routines were performed using Advanced Normalization Tools (ANTs). The Camino Toolkit [26] was used to estimate the diffusion tensors via an unweighted linear least-squares to fit to the log measurements of the diffusion weighted data [27]. To obtain an anatomical labeling for each sample, a multi-atlas approach was used along with a publicly available data set consisting of five manually labeled mouse brains [27]. Diffeomorphic normalization was performed using the mutual information metric provided by ANTs [28] to map each sample's B0 image to each of the five manually labeled brains. The resulting transformations were used to warp the five anatomical label sets into each samples DTI space. Finally, STAPLE [29] was used to merge the multiple label sets to obtain a final set of probabilistic anatomical labels for each sample as shown in Figure 1. Using these methods, each mouse brain was segmented into 39 regions including the ventricles, gray and white matter regions (Figure 1). These probabilistic labels were then used to obtain regional volumes, as well as each region's averaged values for fractional anisotropy (FA), mean diffusivity (MD, ×10^−3^ mm^2^/s), and radial diffusivity (RD). The regional brain volumes were computed and divided by the total brain volume from each individual sample to account for any differences in volumetric differences associated with extraction or fixation of the tissue.

### Statistical analysis

Descriptive statistics were computed for all study variables. Because animals were subjected to social approach and elevated zero maze tests repeatedly over time, a linear mixed effect model was used to analyze the behavioral data which accounts for possible correlation in outcomes collected from the same animal over time. Three factors were considered in this model: group (WT vs NL-3), gender (male vs female) and age/time points (28–29, 48–49 and 68–69 day) as covariates. The three-way interaction effect was first tested using a Wald test followed by testing the two-way interaction effects using likelihood ratio tests. As there was no evidence for interaction in the social sniffing time, the results from the final model that included only the main effects terms are reported. For the anxiety data, the results from a model that contains the main effects terms and an interaction term between group and age are reported.

A linear regression model was used to analyze the cross-sectional volumetric and DTI data that included: group (NL-3 vs WT) as design factor and gender (male vs female), and the actual age (in days), as covariates. The interaction effect was first tested and each region was analyzed separately. Because multiple tests or regression analyses were done for each of the 39 regions for the outcomes of DTI indices and volumes, a false-discovery rate (FDR) was computed to control the type I error and a cut off value of 0.05 was used to determine statistical significance. None of the interaction terms reached statistical significance. Therefore, we reported the results from the model that only included main effects for the three factors. The adjusted mean volume in the significant region was computed from the final model using the predictive average volume of a 50-day male mouse for NL-3 and wild type animal respectively.

Using data from 28 animals that had both behavioral and imaging studies, the correlation between social, anxiety measures and segmented brain volumes were examined by calculating partial correlation coefficient through a linear regression model that have been adjusted for group, gender, and age. A p-value of <0.05 was considered significant. The correlation analysis was performed only on the selected brain regions which showed significant differences in volume between wild type and NL-3 mice. All statistical computations were performed using STATA 12 (StataCorp LP, College Station, Texas, USA) and statistical package for social sciences (SPSS, version 16.0 SPSS, Inc., Chicago, IL, USA).

## Results

**Figure 2:**
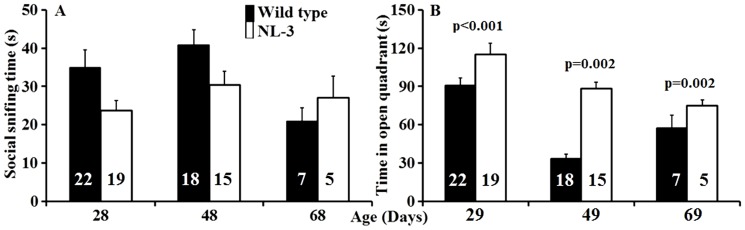
Bar graphs from the NL-3 knock-in and wild type littermates showing average social sniffing time (time spent by the test animal sniffing the stimulus mouse) at 3 different ages (A). Social sniffing test demonstrated significant differences at each time points between wild type and NL-3 mice (p = 0.019) based on a linear mixed effects model adjusting for gender and age and with no significant group interaction. Bar graph showing the average time spent by the animals in the open quadrant of the zero maze test in wild type littermates and NL-3 knock-in mice (B). Longer time spent in the open quadrants is thought to reflect lower anxiety-related behavior. Significant differences in anxiety scores were observed between wild type and NL-3 mice at each time point after adjusting for gender and age using a linear mixed effect model. A significant group interaction was also observed in anxiety score (p<0.05) based on a liner mixed effects model adjusting for gender and age. The numbers inside each bar show the number of animals while the error bars represent SEM.

We found no evidence of 3-way and 2-way interaction effects in social approach behavior (all p-value>0.05). After controlling for gender and age, the NL-3 knock-in animals exhibited lower social preference than the WT animals. On an average, the NL-3 animals spent 8.7 seconds (95% CI: 1 to 15 seconds) less time sniffing the stimulus mouse in comparison to their wild type littermates (p = 0.019) (Figure 2A). On the test of anxiety, the NL-3 mice spent significantly longer time in the open quadrant after controlling for gender and age, indicating lower anxiety (Figure 2B). There was a significant interaction effect between group and age (p = 0.006), with a larger difference in anxiety between NL-3 mice and wild type observed at 49-day time point (83 seconds, 95% CI: 49 to 116 seconds, p = 0.002) and a smaller difference at day 28 (52 seconds, 95% CI: 34 to 70 seconds, p<0.001) and day 69 (56 seconds, 95% CI: 21 to 90 seconds, p = 0.002).

**Figure 3:**
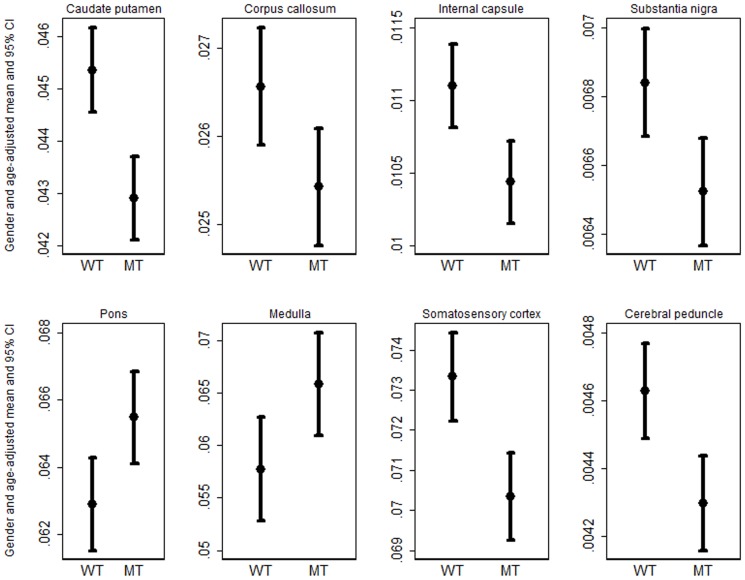
Average and 95% CI of the adjusted brain volume between NL-3 and wild type animals studied from eight out of the 39 regions that showed significant differences in volume.

Raw data on volume and DTI indices at each time points are summarized in Table 1, 2, 3. After FDR correction, no significant interaction was noted and some of the segmented brain regions demonstrated significantly reduced volume in NL-3 knock-in as compared to wild type mice in the adjusted analysis that included age and gender as covariate. However, the gender and age-adjusted volume of the pons and medulla were significantly larger in NL-3 knock-in mice than the wild type littermates (Figure 3). On the other hand, none of the DTI indices demonstrated any significant differences in NL-3 mice compared to wild type from any regions of the brain (Table 1, 2, 3).

**Table 1:** Average ±SD values from DTI indices (FA, MD, and RD) and normalized volume from gray and white matter regions of the brain between wild type (n = 7) and NL-3 (n = 10) mice at 30 days of age.

#	Regions	FA		MD×10^−3^		RD		Normalize brain volume	
		WT	NL-3	WT	NL-3	WT	NL-3	WT	NL-3
1	Lat ventricle	0.36±0.03	0.32±0.03	0.25±0.02	0.26±0.03	0.21±0.02	0.23±0.03	0.20±0.02	0.19±0.02
2	Hippo CA3	0.26±0.01	0.26±0.01	0.25±0.02	0.25±0.02	0.23±0.02	0.23±0.02	0.20±0.02	0.20±0.01
3	Hippo CA1	0.29±0.02	0.29±0.02	0.26±0.01	0.26±0.01	0.23±0.01	0.23±0.01	0.17±0.01	0.19±0.09
4	Hippo dent gyr	0.26±0.02	0.25±0.01	0.26±0.02	0.27±0.01	0.24±0.02	0.25±0.01	0.18±0.02	0.19±0.01
5	Hippo gen	0.30±0.01	0.30±0.01	0.25±0.02	0.25±0.02	0.22±0.02	0.23±0.02	0.18±0.01	0.18±0.01
6	Olfact system	0.25±0.03	0.26±0.03	0.26±0.02	0.25±0.02	0.25±0.02	0.23±0.02	0.83±0.02	0.91±0.01
7	Frontal cortex	0.24±0.02	0.23±0.02	0.26±0.03	0.25±0.03	0.24±0.02	0.23±0.02	0.44±0.01	0.44±0.03
8	Perirh cortex	0.28±0.03	0.26±0.03	0.26±0.02	0.24±0.02	0.25±0.02	0.23±0.02	0.10±0.01	0.10±0.01
9	Entorhi cortex	0.22±0.02	0.20±0.02	0.27±0.01	0.26±0.01	0.25±0.01	0.24±0.01	0.27±0.01	0.27±0.02
10	Cortex general	0.26±0.03	0.25±0.03	0.26±0.02	0.25±0.02	0.24±0.02	0.23±0.01	0.81±0.02	0.78±0.04
11	Caud putamen	0.22±0.01	0.21±0.02	0.25±0.02	0.24±0.02	0.24±0.02	0.23±0.02	0.46±0.02	0.43±0.02
12	B gang general	0.26±0.02	0.25±0.02	0.23±0.02	0.23±0.02	0.21±0.02	0.22±0.02	0.26±0.01	0.25±0.01
13	Corpus callosum	0.51±0.03	0.50±0.03	0.20±0.03	0.19±0.03	0.16±0.02	0.15±0.02	0.27±0.01	0.25±0.02
14	Ant commissure	050±0.03	0.48±0.04	0.22±0.03	0.21±0.03	0.1 6±0.02	0.16±0.02	0.04±0.03	0.04±0.02
15	Lat olfact tract	0.29±0.04	0.31±0.03	0.26±0.03	0.25±0.02	0.24±0.02	0.22±0.02	0.05±0.06	0.05±0.03
16	Internal capsule	0.53±0.01	0.51±0.02	0.21±0.03	0.20±0.03	0.16±0.02	0.16±0.02	0.11±0.01	0.10±0.01
17	Amygdala	0.22±0.02	0.21±0.02	0.25±0.02	0.26±0.02	0.24±0.02	0.24±0.02	0.32±0.01	0.31±0.02
18	Third ventricle	0.25±0.03	0.24±0.03	0.26±0.02	0.27±0.03	0.24±0.01	0.25±0.03	0.09±0.01	0.09±0.01
19	Thalamus	0.33±0.02	0.32±0.02	0.23±0.03	0.22±0.03	0.21±0.03	0.21±0.03	0.44±0.02	0.44±0.02
20	Hypothalamus	0.26±0.02	0.25±0.04	0.23±0.03	0.21±0.02	0.21±0.03	0.20±0.02	0.20±0.01	0.20±0.01
21	Cereb aqueduct	0.24±0.05	0.25±0.04	0.33±0.10	0.26±0.05	0.31±0.10	0.26±0.05	0.29±0.02	0.31±0.01
22	Substantia nigra	0.38±0.03	0.36±0.02	0.22±0.03	0.22±0.02	0.19±0.02	0.19±0.02	0.07±0.01	0.07±0.01
23	Sup+Inf colli	0.25±0.02	0.25±0.01	0.24±0.02	0.23±0.02	0.23±0.02	0.22±0.02	0.36±0.01	0.36±0.02
24	Periaqued Gray	0.25±0.03	0.25±0.02	0.23±0.04	0.22±0.03	0.21±0.03	0.21±0.03	0.12±0.01	0.13±0.01
25	Midbrain general	0.32±0.02	0.31±0.02	0.23±0.03	0.22±0.02	0.21±0.02	0.20±0.02	0.38±0.01	0.38±0.01
26	Cereb general	0.32±0.03	0.31±0.02	0.22±0.02	0.21±0.02	0.20±0.03	0.19±0.02	0.57±0.02	0.58±0.03
27	Fourth ventricle	0.26±0.03	0.25±0.03	0.37±0.05	0.36±0.07	0.35±0.05	0.34±0.08	0.11±0.01	0.11±0.01
28	Pons	0.35±0.03	0.34±0.02	0.27±0.02	0.25±0.02	0.24±0.02	0.22±0.02	0.62±0.02	0.66±0.03
29	Medulla	0.30±0.02	0.30±0.02	0.26±0.03	0.25±0.02	0.24±0.02	0.23±0.02	0.60±0.09	0.65±0.09
30	Optic nerve	0.45±0.06	0.43±0.05	0.27±0.02	0.25±0.02	0.21±0.02	0.21±0.02	0.57±0.03	0.56±0.05
31	Fornix system	0.49±0.05	0.49±0.03	0.22±0.03	0.22±0.02	0.17±0.03	0.16±0.02	0.12±0.01	0.12±0.01
32	Pituitary	0.34±0.09	0.32±0.07	0.41±0.07	0.35±0.09	0.37±0.08	0.33±0.10	0.03±0.01	0.03±0.01
33	Septum	0.29±0.03	0.26±0.03	0.23±0.03	0.23±0.03	0.21±0.02	0.21±0.02	0.08±0.01	0.08±0.01
34	Motor cortex	0.23±0.02	0.23±0.02	0.26±0.03	0.26±0.03	0.25±0.02	0.24±0.03	0.30±0.01	0.30±0.02
35	Somatosen cortex	0.23±0.02	0.22±0.02	0.26±0.02	0.25±0.02	0.25±0.02	0.24±0.02	0.75±0.02	0.71±0.02
36	Auditory cortex	0.25±0.02	0.24±0.02	0.26±0.02	0.25±0.02	0.24±0.02	0.23±0.02	0.13±0.01	0.13±0.01
37	Visual cortex	0.22±0.02	0.21±0.02	0.27±0.02	0.26±0.02	0.25±0.02	0.25±0.02	0.37±0.02	0.36±0.02
38	Cerebellar cortex	0.29±0.04	0.28±0.02	0.24±0.02	0.23±0.01	0.22±0.03	0.21±0.02	0.82±0.04	0.85±0.05
39	Cereb peduncle	0.52±0.03	0.51±0.04	0.27±0.02	0.26±0.02	0.21±0.02	0.21±0.02	0.45±0.01	0.41±0.02

FA  = fractional anisotropy, MD  = mean diffusivity, RD  = radial diffusivity, WT  = wild type. [Lat ventricle  = lateral ventricle; Hippo CA3  =  hippocampus CA3, Hippo CA1  = hippocampus CA1, Hippo dent gyr  = hippocampus dentate gyrus, Hippo gen  = hippocampus general, Olfact system  = olfactory system, Perirh cortex  = perirhinal cortex, Entorhi cortex  =  entorhinal cortex, Caud putamen  = caudate putamen, B gang general  =  basal ganglia general, Ant commissure  = anterior commissure, Lat olfact tract  = lateral olfactory tract, Cereb aqueduct  = cerebral aqueduct, Sup+Inf colli  =  superior and inferior collicus, Periaqued gray  =  periaqueductal gray matter, Cereb general  = cerebellum general, Somatosen cortex  = somatosensory cortex, Cereb peduncle  = cerebral peduncle].

**Table 2:** Average ± SD values from DTI indices (FA, MD, and RD) and normalized volume from gray and white matter regions of the brain between wild type (n  = 9) and NL-3 (n  = 10) mice at 50 days of age.

#	Regions	FA		MD×10^−3^		RD		Normalize brain volume	
		WT	NL-3	WT	NL-3	WT	NL-3	WT	NL-3
1	Lat ventricle	0.34±0.04	0.32±0.04	0.24±0.03	0.26±0.03	0.21±0.02	0.24±0.03	0.20±0.01	0.20±0.02
2	Hippo CA3	0.27±0.02	0.28±0.02	0.24±0.02	0.25±0.02	0.22±0.02	0.23±0.02	0.21±0.02	0.21±0.01
3	Hippo CA1	0.30±0.02	0.30±0.02	0.25±0.01	0.27±0.03	0.23±0.01	0.24±0.02	0.18±0.01	0.17±0.01
4	Hippo dent gyr	0.27±0.03	0.27±0.03	0.26±0.02	0.28±0.02	0.24±0.02	0.26±0.02	0.19±0.02	0.19±0.01
5	Hippo gen	0.30±0.02	0.31±0.03	0.24±0.02	0.25±0.02	0.22±0.02	0.25±0.02	0.19±0.02	0.19±0.01
6	Olfact system	0.24±0.02	0.24±0.02	0.25±0.03	0.26±0.02	0.24±0.03	0.24±0.03	0.80±0.07	0.72±0.04
7	Frontal cortex	0.23±0.01	0.23±0.02	0.24±0.03	0.25±0.03	0.23±0.02	0.24±0.03	0.44±0.02	0.43±0.02
8	Perirh cortex	0.26±0.02	0.26±0.03	0.24±0.03	0.26±0.03	0.22±0.02	0.24±0.02	0.10±0.01	0.10±0.01
9	Entorhi cortex	0.21±0.01	0.21±0.02	0.25±0.03	0.28±0.03	0.24±0.03	0.26±0.03	0.28±0.02	0.28±0.02
10	Cortex general	0.25±0.02	0.26±0.02	0.24±0.02	0.25±0.03	0.23±0.02	0.24±0.01	0.79±0.03	0.77±0.01
11	Caud putamen	0.22±0.02	0.22±0.01	0.24±0.02	0.24±0.02	0.23±0.02	0.23±0.02	0.47±0.02	0.44±0.01
12	B gang general	0.26±0.02	0.26±0.02	0.22±0.02	0.23±0.02	0.21±0.02	0.22±0.02	0.27±0.02	0.26±0.02
13	Corpus callosum	0.51±0.02	0.51±0.03	0.18±0.02	0.19±0.02	0.15±0.01	0.15±0.01	0.27±0.02	0.25±0.02
14	Ant commissure	0.48±0.03	0.46±0.02	0.20±0.02	0.20±0.02	0.1 5±0.01	0.16±0.02	0.036±0.01	0.035±0.01
15	Lat olfact tract	0.28±0.03	0.28±0.02	0.26±0.03	0.25±0.02	0.24±0.02	0.22±0.02	0.05±0.01	0.04±0.01
16	Internal capsule	0.53±0.04	0.52±0.02	0.20±0.02	0.20±0.02	0.15±0.02	0.15±0.02	0.12±0.01	0.11±0.01
17	Amygdala	0.21±0.01	0.22±0.02	0.24±0.04	0.26±0.03	0.23±0.03	0.24±0.02	0.34±0.02	0.32±0.02
18	Third ventricle	0.24±0.04	0.22±0.02	0.26±0.03	0.27±0.03	0.25±0.03	0.26±0.03	0.09±0.01	0.10±0.01
19	Thalamus	0.34±0.04	0.33±0.02	0.21±0.02	0.22±0.02	0.20±0.02	0.20±0.02	0.46±0.03	0.44±0.02
20	Hypothalamus	0.26±0.03	0.26±0.02	0.21±0.03	0.21±0.02	0.20±0.03	0.20±0.02	0.20±0.02	0.20±0.01
21	Cereb aqueduct	0.28±0.09	0.23±0.03	0.28±0.08	0.28±0.05	0.26±0.08	0.27±0.06	0.03±0.01	0.03±0.02
22	Substantia nigra	0.38±0.03	0.37±0.02	0.21±0.02	0.22±0.02	0.19±0.02	0.19±0.02	0.07±0.01	0.07±0.01
23	Sup+Inf colli	0.24±0.03	0.26±0.02	0.22±0.02	0.23±0.02	0.21±0.02	0.22±0.02	0.04±0.03	0.04±0.02
24	Periaqued Gray	0.27±0.06	0.26±0.03	0.20±0.03	0.22±0.02	0.19±0.03	0.21±0.02	0.03±0.01	0.03±0.01
25	Midbrain general	0.33±0.04	0.32±0.02	0.22±0.02	0.23±0.01	0.20±0.02	0.21±0.01	0.40±0.01	0.39±0.01
26	Cereb general	0.32±0.01	0.32±0.01	0.21±0.01	0.21±0.01	0.19±0.01	0.19±0.01	0.56±0.03	0.65±0.02
27	Fourth ventricle	0.25±0.03	0.23±0.03	0.36±0.01	0.39±0.01	0.34±0.01	0.38±0.01	0.10±0.02	0.11±0.01
28	Pons	0.35±0.02	0.34±0.02	0.24±0.02	0.25±0.02	0.21±0.02	0.23±0.02	0.64±0.04	0.66±0.02
29	Medulla	0.33±0.07	0.37±0.11	0.25±0.03	0.26±0.03	0.22±0.03	0.23±0.03	0.52±0.11	0.65±0.11
30	Optic nerve	0.44±0.03	0.43±0.05	0.26±0.02	0.25±0.02	0.21±0.02	0.21±0.02	0.06±0.01	0.06±0.01
31	Fornix system	0.50±0.03	0.49±0.02	0.20±0.02	0.20±0.02	0.15±0.02	0.15±0.01	0.13±0.02	0.13±0.04
32	Pituitary	0.29±0.04	0.28±0.05	0.38±0.01	0.42±0.01	0.36±0.01	0.40±0.01	0.03±0.01	0.03±0.01
33	Septum	0.29±0.03	0.27±0.04	0.22±0.02	0.24±0.02	0.20±0.02	0.22±0.02	0.08±0.01	0.08±0.01
34	Motor cortex	0.21±0.01	0.22±0.02	0.25±0.03	0.25±0.03	0.24±0.03	0.24±0.03	0.32±0.02	0.30±0.02
35	Somatosen cortex	0.21±0.01	0.22±0.01	0.24±0.03	0.25±0.03	0.23±0.03	0.24±0.03	0.76±0.04	0.72±0.02
36	Auditory cortex	0.24±0.01	0.25±0.03	0.24±0.03	0.26±0.03	0.22±0.03	0.24±0.03	0.13±0.01	0.13±0.01
37	Visual cortex	0.20±0.02	0.22±0.02	0.25±0.03	0.25±0.03	0.24±0.03	0.24±0.03	0.36±0.03	0.35±0.03
38	Cerebellar cortex	0.29±0.01	0.27±0.02	0.23±0.01	0.24±0.02	0.21±0.01	0.23±0.02	0.81±0.04	0.84±0.03
39	Cereb peduncle	0.53±0.03	0.52±0.03	0.26±0.03	0.27±0.02	0.20±0.02	0.21±0.03	0.49±0.04	0.44±0.01

FA  = fractional anisotropy, MD  = mean diffusivity, RD  = radial diffusivity, WT  = wild type.

[Lat ventricle  = lateral ventricle; Hippo CA3  =  hippocampus CA3, Hippo CA1  = hippocampus CA1, Hippo dent gyr  = hippocampus dentate gyrus, Hippo gen  = hippocampus general, Olfact system  = olfactory system, Perirh cortex  = perirhinal cortex, Entorhi cortex  =  entorhinal cortex, Caud putamen  = caudate putamen, B gang general  =  basal ganglia general, Ant commissure  = anterior commissure, Lat olfact tract  = lateral olfactory tract, Cereb aqueduct  = cerebral aqueduct, Sup+Inf colli  =  superior and inferior collicus, Periaqued gray  =  periaqueductal gray matter, Cereb general  = cerebellum general, Somatosen cortex  = somatosensory cortex, Cereb peduncle  = cerebral peduncle].

**Table 3:** Average ± SD values from DTI indices (FA, MD, and RD) and normalized volume from gray and white matter regions of the brain between wild type (n  = 8) and NL-3 (n  = 5) mice at 70 days of age.

#	Regions	FA		MD×10^−3^		RD		Normalize brain volume	
		WT	NL-3	WT	NL-3	WT	NL-3	WT	NL-3
1	Lat ventricle	0.31±0.03	0.29±0.02	0.26±0.04	0.30±0.03	0.24±0.04	0.27±0.04	0.20±0.01	0.21±0.02
2	Hippo CA3	0.27±0.04	0.24±0.02	0.25±0.03	0.25±0.02	0.23±0.03	0.24±0.02	0.21±0.01	0.21±0.02
3	Hippo CA1	0.29±0.03	0.27±0.02	0.26±0.02	0.27±0.02	0.24±0.02	0.24±0.02	0.16±0.01	0.17±0.01
4	Hippo dent gyr	0.27±0.04	0.24±0.02	0.28±0.03	0.28±0.03	0.26±0.03	0.26±0.02	0.19±0.02	0.19±0.01
5	Hippo gen	0.30±0.03	0.28±0.02	0.25±0.02	0.25±0.02	0.22±0.02	0.23±0.02	0.18±0.01	0.19±0.01
6	Olfact system	0.23±0.02	0.25±0.02	0.25±0.02	0.25±0.02	0.23±0.02	0.23±0.02	0.81±0.17	0.89±0.15
7	Frontal cortex	0.22±0.02	0.23±0.02	0.25±0.02	0.24±0.02	0.23±0.02	0.23±0.02	0.42±0.02	0.43±0.01
8	Perirh cortex	0.26±0.03	0.26±0.03	0.25±0.02	0.24±0.02	0.24±0.02	0.23±0.02	0.09±0.01	0.10±0.01
9	Entorhi cortex	0.21±0.02	0.20±0.02	0.27±0.01	0.26±0.01	0.25±0.01	0.26±0.01	0.25±0.03	0.26±0.02
10	Cortex general	0.26±0.02	0.25±0.02	0.24±0.01	0.24±0.01	0.23±0.01	0.23±0.01	0.75±0.04	0.75±0.01
11	Caud putamen	0.21±0.02	0.21±0.02	0.24±0.02	0.23±0.02	0.23±0.02	0.22±0.02	0.44±0.02	0.44±0.01
12	B gang general	0.26±0.01	0.25±0.02	0.22±0.02	0.22±0.02	0.21±0.02	0.21±0.02	0.26±0.01	0.26±0.01
13	Corpus callosum	0.53±0.02	0.52±0.02	0.17±0.02	0.17±0.02	0.13±0.02	0.13±0.02	0.26±0.01	0.26±0.01
14	Ant commissure	0.48±0.02	0.48±0.03	0.19±0.02	0.20±0.03	0.1 5±0.01	0.15±0.02	0.04±0.03	0.04±0.03
15	Lat olfact tract	0.26±0.03	0.28±0.03	0.26±0.02	0.25±0.02	0.24±0.02	0.22±0.01	0.05±0.01	0.05±0.01
16	Internal capsule	0.53±0.03	0.51±0.03	0.19±0.03	0.18±0.03	0.14±0.02	0.14±0.02	0.11±0.01	0.10±0.02
17	Amygdala	0.21±0.02	0.21±0.02	0.25±0.01	0.25±0.02	0.24±0.01	0.24±0.02	0.32±0.02	0.32±0.02
18	Third ventricle	0.21±0.03	0.20±0.03	0.28±0.03	0.27±0.04	0.27±0.03	0.26±0.04	0.10±0.04	0.10±0.01
19	Thalamus	0.34±0.06	0.31±0.01	0.21±0.04	0.21±0.02	0.19±0.04	0.20±0.02	0.44±0.01	0.42±0.02
20	Hypothalamus	0.24±0.02	0.24±0.03	0.19±0.01	0.21±0.03	0.18±0.01	0.20±0.03	0.20±0.01	0.20±0.01
21	Cereb aqueduct	0.22±0.04	0.22±0.03	0.39±0.07	0.29±0.07	0.38±0.08	0.28±0.07	0.30±0.03	0.31±0.04
22	Substantia nigra	0.38±0.04	0.36±0.02	0.20±0.03	0.21±0.03	0.21±0.02	0.22±0.01	0.07±0.01	0.07±0.01
23	Sup+Inf colli	0.25±0.03	0.24±0.01	0.23±0.02	0.23±0.02	0.22±0.02	0.22±0.02	0.35±0.02	0.35±0.01
24	Periaqued Gray	0.25±0.05	0.23±0.01	0.20±0.03	0.22±0.03	0.19±0.03	0.21±0.03	0.13±0.01	0.13±0.01
25	Midbrain general	0.32±0.04	0.30±0.02	0.22±0.03	0.22±0.03	0.20±0.03	0.20±0.02	0.38±0.02	0.38±0.01
26	Cereb general	0.31±0.01	0.31±0.02	0.21±0.01	0.22±0.01	0.21±0.01	0.22±0.02	0.58±0.03	0.58±0.02
27	Fourth ventricle	0.22±0.02	0.23±0.02	0.44±0.06	0.38±0.05	0.43±0.06	0.38±0.05	0.11±0.01	0.12±0.02
28	Pons	0.33±0.02	0.33±0.03	0.25±0.02	0.24±0.03	0.22±0.02	0.22±0.02	0.64±0.02	0.66±0.03
29	Medulla	0.29±0.02	0.29±0.02	0.26±0.02	0.24±0.02	0.23±0.02	0.22±0.02	0.61±0.11	0.69±0.04
30	Optic nerve	0.41±0.02	0.41±0.04	0.27±0.02	0.24±0.03	0.23±0.02	0.20±0.02	0.06±0.01	0.06±0.01
31	Fornix system	0.51±0.03	0.49±0.03	0.19±0.03	0.19±0.03	0.14±0.02	0.15±0.02	0.13±0.01	0.13±0.01
32	Pituitary	0.28±0.05	0.27±0.06	0.19±0.03	0.19±0.03	0.14±0.02	0.15±0.02	0.03±0.01	0.03±0.01
33	Septum	0.26±0.03	0.24±0.03	0.22±0.03	0.22±0.02	0.20±0.03	0.21±0.02	0.08±0.01	0.08±0.01
34	Motor cortex	0.22±0.01	0.21±0.02	0.24±0.02	0.25±0.01	0.28±0.02	0.23±0.01	0.29±0.02	0.29±0.01
35	Somatosen cortex	0.21±0.02	0.20±0.02	0.24±0.02	0.24±0.01	0.23±0.01	0.23±0.01	0.71±0.02	0.68±0.02
36	Auditory cortex	0.24±0.03	0.22±0.01	0.25±0.02	0.24±0.01	0.23±0.02	0.23±0.01	0.12±0.01	0.12±0.01
37	Visual cortex	0.20±0.02	0.19±0.01	0.25±0.01	0.24±0.01	0.24±0.01	0.23±0.01	0.34±0.01	0.32±0.01
38	Cerebellar cortex	0.27±0.01	0.27±0.02	0.25±0.02	0.24±0.01	0.24±0.02	0.23±0.01	0.84±0.04	0.83±0.04
39	Cereb peduncle	0.54±0.04	0.51±0.04	0.24±0.04	0.26±0.03	0.18±0.04	0.21±0.02	0.45±0.01	0.44±0.01

FA  = fractional anisotropy, MD  = mean diffusivity, RD  = radial diffusivity, WT  = wild type.

[Lat ventricle  = lateral ventricle; Hippo CA3  =  hippocampus CA3, Hippo CA1  = hippocampus CA1, Hippo dent gyr  = hippocampus dentate gyrus, Hippo gen  = hippocampus general, Olfact system  = olfactory system, Perirh cortex  = perirhinal cortex, Entorhi cortex  =  entorhinal cortex, Caud putamen  = caudate putamen, B gang general  =  basal ganglia general, Ant commissure  = anterior commissure, Lat olfact tract  = lateral olfactory tract, Cereb aqueduct  = cerebral aqueduct, Sup+Inf colli  =  superior and inferior collicus, Periaqued gray  =  periaqueductal gray matter, Cereb general  = cerebellum general, Somatosen cortex  = somatosensory cortex, Cereb peduncle  = cerebral peduncle].

Correlation between the brain regions which demonstrated significant differences in volume (8 regions) and behavioral data (social sniffing time and elevated zero maze tests) was also performed. No significant correlation between these brain regions and social sniffing time (partial correlations ranged from −0.014 to 0.313, all p>0.05) was observed. However, a significant correlation between the volume of internal capsule (partial correlation  = −0.447, p = 0.025) and medulla (partial correlation  = 0.407, p = 0.043) and elevated zero maze test score was observed after accounting for strain, gender and age as covariates.

## Discussion

We observed significantly lower volume from 6 brain regions and higher volume from the pons and medulla in NL-3 knock-in mice compared to the wild type littermates after considering age and gender as covariates. These volumetric differences were found at all the three stages of development (juvenile, peri-pubescent and early adult) indicating that the NL-3 knock-in mutation causes alterations in these brain areas that are developmentally stable. While volumetric differences were observed, no significant differences in DTI parameters were observed between NL-3 knock-in and WT mice. These results are in agreement with a previously published MRI study on a smaller sample of 108-day-old male NL-3 knock-in mice [3]. Additionally, NL-3 knock-in mice demonstrated significantly reduced social approach and anxiety-related behavior compared to wild type litter-mates after considering age and gender as covariates.

The neuroligin and neurexin genes have been recognized in human autism association studies [7], [30]. Jamain et al. [7], reported an inherited mutation in NL-3 gene within a highly conserved region of the gene in two male siblings, one with autism and severe intellectual disabilities and seizures and the other with Asperger syndrome. To gain insights into the possible mechanisms of ASDs, this genetic mutation was introduced in the R451C substitution mutation into a mouse to create the NL-3 R451C knock-in mouse model associated with autism [6]. However, conflicting results were reported from the behavioral studies on this model, with one group [6] reporting behavioral abnormalities and other group [8] not observing any autism relevant behavioral abnormalities in this model. We also observed significantly lower social approach behavior in NL-3 mice compared to wild type litter mates. While our findings may seem to be similar to the observations made by Tabuchi et al [6] and in contrast to the study by Chadman et al [8], it should be noted that our experiments and analysis were somewhat distinct from both studies in that we conducted Social Approach testing at multiple ages, and found the reduction in social approach behavior to be more striking at earlier ages whereas the other studies conducted Social Approach Testing only in adult mice.

MRI has been used for examining both volume changes and white matter structural integrity in patients with ASD [9], [31] as well as in mouse models of ASD [2], [3], [12], [14]. We observed significantly lower volume from the caudate putamen, substantia nigra, somatosensory cortex, corpus callosum, internal capsule and cerebral peduncles along with significantly higher volume from the pons and medulla in NL-3 knock-in mice compared to wild type litter mates. The volumetric differences observed in NL-3 mice in the current study suggest that the microstructural abnormality in this model is diffuse and affects several regions of the brain. A previous MRI study on NL-3 mice brains also reported lower volume in major white matter regions especially the corpus callosum, internal capsule and cerebral peduncles [3]. Specific structures, like the corpus callosum, have long been implicated in human autism and reduced corpus callosum volume has been reported in various mouse models of autism [3], [12], [20], suggesting decreased interhemispheric connectivity in these models. The corpus callosum enables communication between the two hemispheres and reduced corpus callosum size may indicate white matter deficits that result in impaired cortical connectivity [32]. The lower volume of the other white matter regions (internal capsule and cerebral peduncle) in NL-3 mice suggest a plausible neural basis for disrupted systems-level connectivity in this model [33]. These white matter regions have also been reported to be smaller in size in mouse models relevant to autism including NL-3 knock-in [3] and the integrin β3 [34] knockout models. Human autism MRI studies have also reported reduced internal capsule and cerebral peduncle volumes [35], however, many other studies indicate enlargement of brain and subcortical regions in ASD.

ASD comprises a set of neurodevelopmental disorders affecting socio-communicative behavior along with abnormalities in the sensorimotor skill learning, oculomotor control, and executive functioning. Some of these impairments may be related to abnormalities in the caudate nuclei, substantia nigra and somatosensory cortex as these regions have been reported to be abnormal in ASD. One of the core behavioral symptoms in autism is restricted and repetitive behaviors, and these behaviors have often been thought to be associated with the caudate putamen or striatum [36]. Structural imaging studies have also reported volumetric differences in the caudate putamen and cortex of humans with ASD [10], [37]. Similar to a previous report, our results of reduced volume from these regions indicate that genetic differences due to the NL-3 knock-in mutation might be responsible for the volumetric changes in this model [3].

The link between neuroanatomical findings and behavioral symptoms is vital for understanding the role of structural changes in the etiology of ASD. Investigations of structural measures of the corpus callosum and other white matter regions have reported decreased size along with under connectivity associated with behavioral abnormalities in ASDs [12], [20]. Overall reduction of whole brain volume in NL-3 mouse has also been reported previously using ex vivo MRI [18].

While our results of reduced volume in both gray and white matter of NL-3 knock-in agree with previous published reports in human ASD as well as in mouse models relevant to ASD, it is worth noting that there is significant heterogeneity in the literature with respect to volumetric differences in cortical thickness and morphology in ASD, with at times seemingly contradictory results, depending on the age, IQ, and clinical severity of the study population [38]. One potential reason for the variability in volumetric differences in human ASD studies could be due to its etiological and clinical heterogeneity. In the current study, we also observed these volumetric differences, which occurred at all three developmental stages of the animal, confirming that the differences were due to genetic mutation that stayed throughout the life span of the animal. Reduced brain volume from several gray and white matter regions of the brain in the NL-3 mice suggest that volumetric MRI can be used to better understand the biological basis of behavioral changes seen in this genetically defined model of ASD.

DTI is sensitive to the integrity of white matter and provides a better understanding of the neuroanatomical abnormalities in ASD. Several DTI studies have shed light on microstructural integrity of the white matter and developmental abnormalities in various brain regions in ASD in human [10], [11], [15]–[17], [35], [38] as well as mouse models relevant to ASD [2], [12], [14], [34]. DTI has also been proposed as a surrogate imaging biomarker in the diagnosis of ASD in humans [35], [39]–[41]. It has also been reported that DTI is not only sensitive for detection of microstructural abnormalities but also correlates with behavioral abnormalities in the BALB/cJ mouse model relevant for ASD [12]. However, recently Koldewyn et al. [42], reported no differences in major white matter structures between autistic children and healthy controls except in right inferior longitudinal fasciculus using DTI. The authors of this study suggested that the abnormalities in FA of ASD subjects reported in previously published studies may probably be due to an artifact from differential head motion during the scan of autistic children [42], [43] raising concerns about the validity of DTI as an imaging biomarker for ASD. We also did not find any significant differences in DTI parameters between NL-3 knock-in and wild type animals in the current study, which may be due to the fact that the genotypic differences in NL-3 mutation may not have had a sufficient effect on water diffusion to affect DTI parameters in this model.

We did not observe any significant correlation between brain volumes and social behavior of the mice. However a significant correlation between volume from internal capsule and medulla with anxiety score was observed in these animals. These preliminary studies in a small set of behavior assays used for assessing rodent behaviors indicate that imaging and behavior tests may act as independent parameters for the diagnosis of ASD in mouse models. Although more studies are needed, we believe that developmental studies involving behavioral and imaging assays in a genetically defined model of ASD, as performed in the current study, will aid in further understanding the biology of ASD in relevant mouse models.

Our results should be interpreted with caution in light of limitations of the study, which include the following: 1) we have used only two behavioral assays - the social approach test and the elevated zero maze test, however other behavioral assays including studies of repetitive behaviors, ultrasonic vocalizations, social memory, open field locomotion, Morris water maze spatial learning and acquisition could provide a more detailed interpretation of behavioral phenotypes relevant to ASD as reported in previous studies [6], [8], [18]. 2) Another limitation is that we did not perform any histological studies on brain tissue to confirm the differences in volume of the various regions in the NL-3 mice. Histological measures of volume are time consuming and are confounded by the variabilities induced in tissue sectioning and fixation and thus image based segmentation, as reported here, is believed to provide more accurate volume estimates of brain anatomy. Nevertheless, future studies correlating histological assessments with MRI are warranted.

In summary, reduced volume of several white and gray matter areas in the brains of NL-3 knock-in mice suggests that these structural changes are induced by the mutation in the NL-3 gene. Future imaging and behavior studies on NL-4 knock-in and knockout models, which have been suggested to be more relevant to ASD [44], may help in establishing the role of neuroligins in ASD.

## Supporting Information

Table S1 Distribution of animals used in the study.
(DOCX)
