# Supplementary material for: High Resolution Magnetic Resonance Imaging for Characterization of the Neuroligin-3 Knock-in Mouse Model Associated with Autism Spectrum Disorder
Source: PLoS One. 2014 Oct 9;9(10):e109872. doi: 10.1371/journal.pone.0109872 (PMC4192590; doi:10.1371/journal.pone.0109872)
Supplement: Table S1 — Distribution of animals used in the study. (DOCX) [file pone.0109872.s001.docx]

**Supplementary Table 1**: Distribution of animals used in the study.

| Social approach and elevated zero maze test | | | | |
| --- | --- | --- | --- | --- |
| Time point  (age in days) | Wild type | | NL-3 | |
|  | Male | Female | Male | Female |
| 28 | 19 | 3 | 13 | 6 |
| 48 | 15 | 3 | 11 | 4 |
| 68 | 5 | 2 | 5 | 0 |
| MRI | | | | |
| 30 | 4 | 3 | 5 | 5 |
| 50 | 6 | 3 | 6 | 4 |
| 70 | 6 | 2 | 4 | 1 |
